# *Coffea canephora* Peptides in Combinatorial Treatment with Fluconazole: Antimicrobial Activity against Phytopathogenic Fungus

**DOI:** 10.1155/2018/8546470

**Published:** 2018-07-10

**Authors:** Gabriela C. V. Bard, Gabriel B. Taveira, Thaynã A. M. Souza, Érica O. Mello, Sávio B. Souza, Alessandro C. Ramos, André O. Carvalho, Lídia S. Pereira, Umberto Zottich, Rosana Rodrigues, Valdirene M. Gomes

**Affiliations:** ^1^Centro de Biociências e Biotecnologia, Universidade Estadual do Norte Fluminense, 28013-602 Campos dos Goytacazes, RJ, Brazil; ^2^Centro de Ciências e Tecnologias Agropecuárias, Universidade Estadual do Norte Fluminense, 28013-602 Campos dos Goytacazes, RJ, Brazil; ^3^Centro de Ciências da Saúde, Universidade Federal de Roraima, Boa Vista, RR, Brazil

## Abstract

The objective of the present study was to evaluate the antimicrobial activity of the *Cc*-LTP2 and *Cc*-GRP peptides isolated from *Coffea canephora* seeds and their possible synergistic activity with the azole drug fluconazole and characterize their mechanisms of action on cells of pathogenic fungi. *Cc*-LTP2 and *Cc*-GRP alone or in combination with 20 *µ*g/mL of fluconazole were evaluated for their antimicrobial activity on the fungus *Fusarium solani*, and the effects of these peptides on the permeability of membranes and the induction of oxidative stress were determined. Our results show that these peptides at a concentration of 400 *µ*g/mL combined with 20 *µ*g/mL of fluconazole were able to inhibit the growth of the tested fungi, promote changes in their growth pattern, permeabilize the membrane, and induce reactive oxygen species (ROS). Some of these results were also observed with the peptides alone or with fluconazole alone, suggesting that the peptides act synergistically, promoting the potentiation of antimicrobial action. In this study, it was shown that *Cc*-LTP2 and *Cc*-GRP in combination with fluconazole were able to inhibit the growth of the fungus *F. solani*, to promote permeabilization of its membrane, and to induce the production of ROS, suggesting a combinatorial activity between the peptides and fluconazole.

## 1. Introduction

Plant diseases caused by viruses, bacteria, and fungi affect crops and are responsible for decreasing the quality of agricultural products and additionally can cause significant yield losses [1]. Plant pathogenic fungi are the main organisms responsible for diseases in plants and act as obligatory or facultative parasites [2, 3], representing approximately 70% of these infections. The genus *Fusarium* encompasses some of the major pathogens of agronomic importance that are responsible for losses that reach billions of dollars [4]. Most representatives of this genus are saprophytes and live in soil, plants, and other organic substrates and are especially common in tropical and temperate regions. Species in this genus are responsible for mycotoxin production that not only affects harvests and productivity but also is harmful to animals and humans [5]. Another great concern about the *Fusarium* genus is the broad number of *Fusarium* species that besides being able to infect plants are also able to cause opportunistic infection in animals and humans [6]. The number of reported cases of human infection with *Fusarium* spp. is increasing worldwide due to the vast number of susceptible and immunocompromised patients that are being diagnosed with improved detection methods [6, 7]. Seven main opportunistic *Fusarium* species can cause infection in humans, namely, *Fusarium solani*, *Fusarium oxysporum*, *F. incarnatum-equiseti*, *F. fujikuroi*, *F. chlamydosporum*, *F. dimerum*, and *F. sporotrichioides*. Particularly, the species belonging to *F. solani* and *F. oxysporum* have been reported infecting humans worldwide [4, 8, 9]. One of the main concerns about these infections is the relative insensibility of *Fusarium* to the antifungal compounds available at market, including the azole class [10, 11].

Today, the control of fungal diseases is mostly achieved using chemical fungicides. However, the use of such products may result in a negative impact on the environment and human health. In addition, their prolonged use may result in the selection of resistant phytopathogenic fungi, thus reducing the long-term efficiency of these fungicides, making them increasingly less effective [12, 13].

To combat the increasing resistance in phytopathogenic fungi and reduce the negative impacts on human health and on the environment caused by classical fungicides, numerous strategies have been employed. The use of artificially selected plants with resistant genotypes, production of resistant transgenic varieties, and biological control using other microorganisms are some of the most used strategies. Therefore, the discovery of new antifungal agents, particularly those produced by the plants themselves, for example, proteins and peptides, has also been the focus of many studies in cultivated plants. Antimicrobial proteins and peptides isolated from cultivated or wild plants that are involved in plant defense mechanisms provide the possibility of using these molecules to develop new strategies to control diseases caused by phytopathogenic fungi [14–16].

Antimicrobial peptides (AMPs) are evolutionarily ancient molecules considered part of the innate immune system of many species and are described as components of both constitutive defense and induced defense [17–19]. Plant AMPs are small molecules with molecular weights less than 10 kDa that are rich in cysteine and are amphipathic, giving them the ability to interact with the membranes of target microorganisms. Among plant AMPs are the lipid transfer proteins (LTPs), a group composed of two superfamilies, LTP-1 and LTP-2, with molecular weights of approximately 9 kDa and 7 kDa, respectively [20]. Some LTPs have been reported to inhibit the growth of bacteria [21], phytopathogenic fungi, and yeasts [22, 23].

Another important group of plant-derived proteins and peptides is the glycine-rich proteins (GRPs), which include sequences rich in repetitive glycine domains. More recently, some representatives of this class have demonstrated antimicrobial activity and are capable of inhibiting the growth of phytopathogenic fungi, yeasts [24–26], and Gram-negative bacteria [24].

In a previous report, we had isolated two antifungal peptides from coffee seeds: a GRP, named “*Cc*-GRP,” that showed activity against phytopathogenic fungi and yeast from *Candida* genus, and an LTP, named “*Cc*-LTP2,” that similar to *Cc*-GRP, promoted the permeability of the fungal membrane and induced the production of reactive oxygen species (ROS) [27, 28]. Fluconazole, in combination with AMPs, has shown promising antifungal activity against important fungal pathogens such as yeasts of the *Candida* genus and *Cryptococcus neoformans* [29–31]. Therefore, in this study, we investigated whether the *Cc*-GRP and *Cc*-LTP2 peptides could act synergistically with the commercial drug fluconazole to enhance their effects.

## 2. Materials and Methods

### 2.1. Microorganisms

*Fusarium solani* (4014) was cultured in Sabouraud agar and preserved in the Laboratório de Fisiologia e Bioquímica de Microrganismos (LFBM), Universidade Estadual do Norte Fluminense Darcy Ribeiro (UENF), Campos dos Goytacazes, RJ, Brazil. The phytopathogenic fungus was maintained in Sabouraud 2% glucose agar (Merck).

### 2.2. *Cc*-GRP and *Cc*-LTP2

Extraction and purification of the *Cc*-GRP and *Cc*-LTP2 from *Coffea canephora* seeds were performed as described by Zottich et al. [26] and Bard et al. [28], respectively.

### 2.3. Combinatorial Treatment Assay and Determination of Dry Weight

To determine the synergistic activities, we combined fluconazole (FLC) (Sigma-Aldrich) with *Cc*-GRP and/or *Cc*-LTP2.

Initially, fungal spores (1 × 10^4^ spores/mL) were incubated in Sabouraud broth (Merck Millipore). The assay was performed in 96-well microplates (Nunc) at 30°C for 48 h with the final volume adjusted to 200 *μ*L. Control cells were (1) grown in the absence of *Cc*-GRP, *Cc*-LTP2 (400 *µ*g/mL each), or FLC (20 *µ*g/mL, solubilized in 5% dimethyl sulfoxide (DMSO), which is not a DMSO toxic concentration for *F. solani*), (2) grown in the presence of FLC, or (3) grown in the presence of *Cc*-GRP or *Cc*-LTP2. Herein, we define synergism as the action of the antimicrobial peptides (AMPs) combined with FLC that causes an enhanced diminution in the dry weight of the microorganism compared with the dry weight of a single substance. After the assay of dry weight, the cells (controls and tests) were analyzed by a differential interference contrast (DIC) optical microscope (AxioVision 4, Zeiss). The data were obtained from triplicate experiments. The data were evaluated using a one-way ANOVA. Mean differences at *p* < 0.05 were considered to be significant. All statistical analyses were performed using the GraphPad Prism software (version 5.0 for Windows).

### 2.4. Plasma Membrane Permeabilization Assay

Permeabilization of the fungal plasma membrane was measured by Sytox Green uptake according to the methodology described by Thevissen et al. [32], with some modifications. In brief, the pathogenic fungus *Fusarium solani* was incubated with 400 *µ*g/mL of *Cc*-GRP or *Cc*-LTP2 separately and in combination with 20 *µ*g/mL of FLC for 24 h. After this time, a 100 *μ*L aliquot of cell suspension was incubated with 0.2 *μ*M of Sytox Green (Molecular Probes, Invitrogen) in a 1.5 mL microcentrifuge tube for 30 min at 25°C under constant agitation. The cells were analyzed with a DIC optical microscope (AxioVision 4, Zeiss) equipped with a fluorescent filter set for fluorescein detection (excitation wavelength 450–490 nm; emission wavelength 500 nm).

### 2.5. Determining the Induction of Intracellular ROS in Fungal Cells

To evaluate whether the mechanism of action of *Cc*-GRP and *Cc*-LTP2 involves the induction of oxidative stress, the fluorescent probe 2,7-dichlorofluorescein diacetate (H_2_DCFDA) (Calbiochem-EMD) was used to measure intracellular ROS following the methodology described by Mello et al. [33]. The fungus *F. solani* was incubated with 400 *µ*g/mL of *Cc*-GRP or *Cc*-LTP2 separately and in combination with 20 *µ*g/mL of FLC for 24 h at 30°C, and after this incubation, an aliquot of 50 *μ*L for each combination was incubated with 200 *μ*M of H_2_DCFDA in 1.5 mL microcentrifuge tubes for 1 h at 25°C under constant agitation at 500 rpm. The cells were analyzed with a DIC optical microscope (AxioVision 4, Zeiss) equipped with a fluorescent filter set for fluorescein detection (excitation wavelength 450–490 nm; emission wavelength 500 nm). The experiments were performed in triplicate.

### 2.6. Measurements of H^+^ Flux and Current Using an Anion-Selective Vibrating Probe System

*Fusarium solani* was grown in 40 mL of Sabouraud broth for 24 h at 30°C and 0.75 rpm. After 48 h, the cellular suspension was filtered through gauze to prevent the passage of mycelia that was in solution together with the conidia. Next, an aliquot of 1 *µ*L of conidial suspension was transferred to the centre of a Petri dish (40 × 10 mm) containing 1 mL of Sabouraud agar and grown for 24 h at 30°C. After this growth period, 2 mL of Sabouraud broth was gently added into the Petri dish, and in order to determine the extracellular voltage differences, proton flux, and cell surface pH, measurements of the H^+^ flux were performed by an H^+^ selective vibrating probe.

A detailed description of the experiment with the ion-selective vibrating probe technique used in this study is described in previous works [34–36]. Micropipettes composed of 1.5 mm borosilicate glass capillaries were stretched and treated with dimethyl dichlorosilane (Sigma-Aldrich). After silanization, the microelectrodes were backloaded with electrolyte solution (15 mM KCl and 40 mM KH_2_PO_4_, pH 6.0 for H^+^, from a 15 to 20 mm microelectrode column) and then frontloaded with the respective ion-selective liquid exchange cocktail (Fluka, from a 20 to 25 *µ*m electrolyte column). An Ag/AgCl wire electrode holder (World Precision Instruments) was inserted into the back of the microelectrode for electrical contact with the bathing solution. The ground electrode was used as a dry reference (DRIREF-2, World Precision Instruments) and was inserted into the sample bath. The microelectrodes were calibrated by measuring the background signal at the beginning and the end of each experiment using standard solutions covering the experimental range of each ion. Both the slope and intercept of the calibration line were used to calculate the respective ion concentration from the mV values measured during the experiments.

The treatments with *Cc*-LTP2 (400 *µ*g/mL) and *Cc*-LTP2 (400 *µ*g/mL) + FLC (20 *µ*g/mL) were performed in *F. solani* colonies by determination of H^+^ flux at the hyphal tip for a minimum of 5 min or until reach the steady state (assay was done twice and in triplicate, *n*=6). After that, a background reference was taken, and H^+^ flux was recorded again.

### 2.7. Statistical Analysis

The experiments were conducted in a completely randomized design and analyzed statistically through two-way ANOVA. When a factor or any interactions between factors were considered statistically significant, grouped comparisons were performed through a Tukey test at *p* ≤ 0.05. All analyses were conducted using the GraphPad Prism 7.0 program, using a 5% level of significance for hypothesis testing.

## 3. Results

### 3.1. Effect of the Combination of *Cc*-LTP2 and *Cc*-GRP with Fluconazole (FLC) on the Growth of *F. solani*

**Figure 1:**
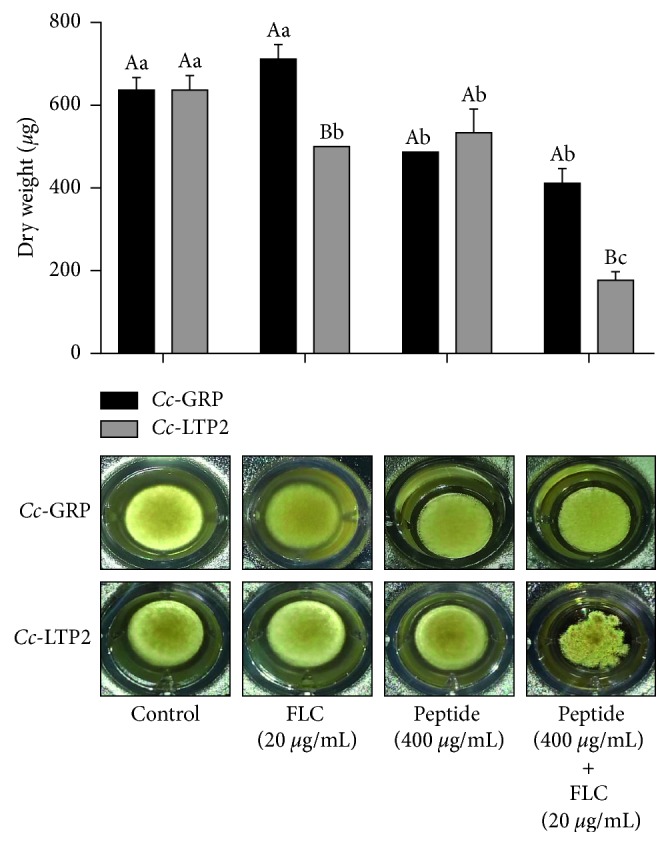
Dry weight of the fungus *F. solani* and photographs of growth on microplates in the presence of *Cc*-LTP2, FLC, or *Cc*-LTP2 plus FLC, and *Cc*-GRP, FLC, or *Cc*-GRP plus FLC after 48 h of treatment. The data were analyzed by two-way ANOVA combined with Tukey's test. For each peptide, bars followed by the same uppercase letter, in different treatments (*Cc*-GRP and *Cc*-LTP2), are not significantly different by Tukey's test at *p* < 0.05. For each treatment (control, FLC, peptide, and peptide plus FLC), bars followed by the same lowercase letter are not significantly different at *p* < 0.05 (*n*=3).

The effect of *Cc*-LTP2 and *Cc*-GRP on the growth of *F. solani* was evaluated by determining the dry weight of the fungus grown in the absence (control) and in the presence of *Cc*-LTP2 and *Cc*-GRP separately and in combination with FLC (Figure 1). The analysis of the dry weight showed that *Cc*-LTP2 (400 *µ*g/mL), when combined with FLC (20 *µ*g/mL), induced a significant reduction in the fungal dry weight compared to the control. However, the same was not observed for the *Cc*-LTP2 and FLC when tested separately. The significant reduction in the growth and the change in the fungal growth pattern were also observed in the photos of the fungus (Figure 1).

**Figure 2:**
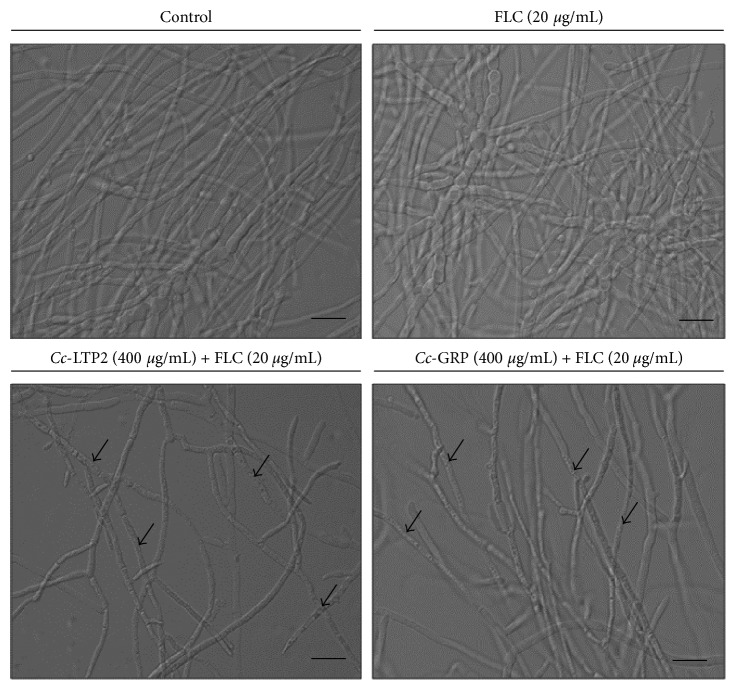
Optical microscopy of the fungus *F. solani* growing in the presence of FLC, *Cc*-LTP2 plus FLC, or *Cc*-GRP plus FLC (bars: 20 *µ*m). Arrows show collapsed and disorganized cytoplasm.

*Cc*-GRP (400 *µ*g/mL), in combination with FLC (20 *µ*g/mL), caused a significant reduction in the dry weight of the fungus compared to controls, indicating the inhibition of *F. solani* fungus. *Cc*-GRP alone (400 *µ*g/mL) also caused a significant reduction in the dry weight of the fungus. Changes in the growth pattern of *F. solani* on the bottom well by direct visualization were not observed, both in the absence (control) and in the presence of *Cc*-GRP (400 *µ*g/mL) and FLC (20 *µ*g/mL), separately or in combination (Figure 2).

Figure 2 shows the effect of FLC (20 *µ*g/mL) on the growth of *F. solani*, both separately and in combination as follows: *Cc*-LTP2 (400 *µ*g/mL) + FLC (20 *µ*g/mL) and *Cc*-GRP (400 *µ*g/mL) + FLC (20 *µ*g/mL). The cells grown in the presence of FLC separately showed no change in their quantity or morphology and were similar to control cells. The cells treated with the two combinations showed a reduction in the amount of hyphae, as well as collapsed and disorganized cytoplasm compared to the control. This result corroborates with the results of the dry weight assay (Figure 1). These results suggest that the *Cc*-LTP2 and *Cc*-GRP peptides acted synergistically with FLC to inhibit the growth of the fungus *F. solani* and promote morphological changes in the fungal cells.

### 3.2. Permeabilization of *Fusarium solani* Plasma Membrane

**Figure 3:**
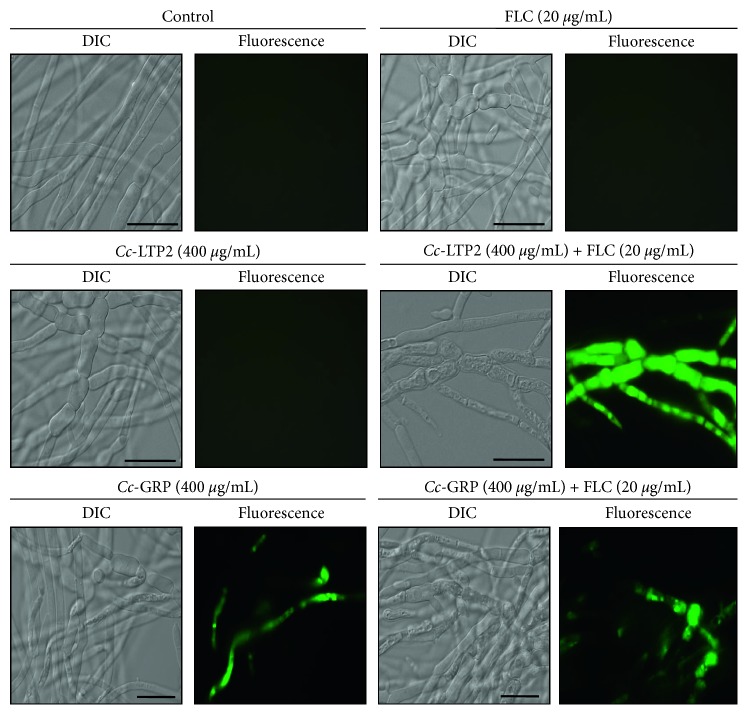
Membrane permeabilization assay. Photomicrography of *F. solani* cells by fluorescence microscopy using the fluorescent probe Sytox Green. The cells were treated with FLC, *Cc*-LTP2, *Cc*-LTP2 plus FLC, *Cc*-GRP, or *Cc*-GRP plus FLC for 24 h and then assayed for omembrane permeabilization. Control cells were treated only with Sytox Green. Bars: 20 *µ*m.

The *Cc*-LTP2 and *Cc*-GRP peptides, separately and in combination with FLC, were tested for the ability to permeabilize the membrane of the fungus *F. solani*. In Figure 3, it can be seen that the fungus grown in the presence of FLC (20 *µ*g/mL) and *Cc*-LTP2 (400 *µ*g/mL) separately, as well as the control, showed no labelling for the dye Sytox Green; that is, they were not able to permeabilize the membrane of the fungus. However, the fungus grown in the presence of the combination *Cc*-LTP2 (400 *µ*g/mL) + FLC (20 *µ*g/mL) presented markings, showing that the joint action of these substances was able to promote structural changes in the plasma membrane of this fungus, promoting its permeabilization. The *Cc*-GRP peptide (400 *µ*g/mL), both separately and in combination with FLC (20 *µ*g/mL), induced permeabilization of the *F. solani* cells.

### 3.3. Oxidative Stress Assay

**Figure 4:**
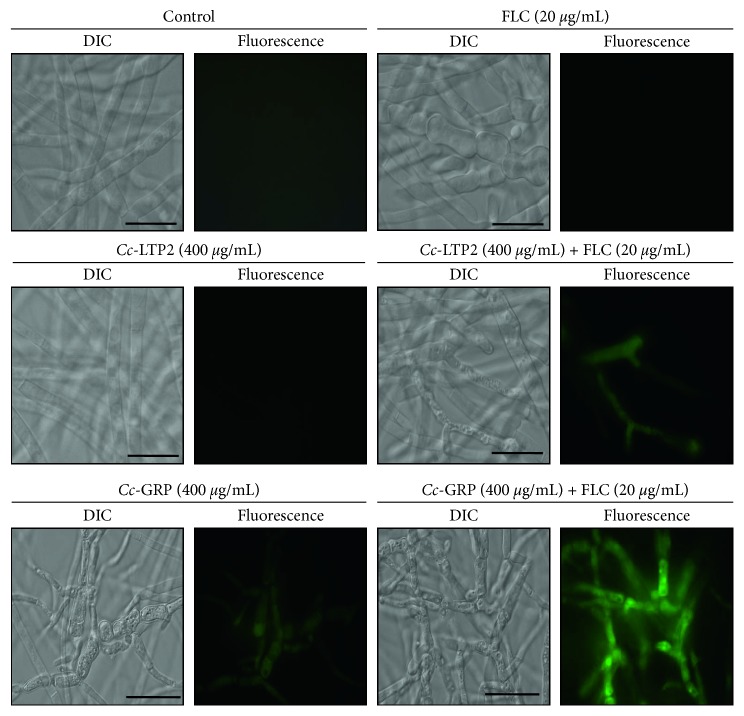
Oxidative stress assays. Photomicrographs of *F. solani* cells after oxidative stress assays by fluorescence microscopy using the probe 2,7-dichlorofluorescein diacetate (H_2_DCFDA). The cells were treated with FLC, *Cc*-LTP2, *Cc*-LTP2 plus FLC, *Cc*-GRP, or *Cc*-GRP plus FLC for 24 h and then assayed for oxidative stress. Control cells were treated only with H_2_DCFDA. Bars: 20 *µ*m.

To assess whether *Cc*-LTP2 and *Cc*-GRP separately and in combination with FLC were able to induce the endogenous production of ROS, the fungus grown in the absence (control) or in the presence of *Cc*-LTP2 and *Cc*-GRP, separately and in combination with FLC, was incubated for 15 min with the H_2_DCFDA dye and then analyzed with a fluorescence optical microscope. As seen in Figure 4, the fungus grown in the presence of *Cc*-LTP2 and *Cc*-GRP separately as well as the control showed no fluorescence. *Cc*-LTP2 (400 *µ*g/mL) when combined with FLC (20 *µ*g/mL) was able to induce endogenous production of ROS. *Cc*-GRP, both separately and in combination with FLC (20 *µ*g/mL), was also able to induce the endogenous production of ROS.

### 3.4. Analysis of H^+^ Flow Using an Ion-Selective Vibrating Electrode System

**Figure 5:**
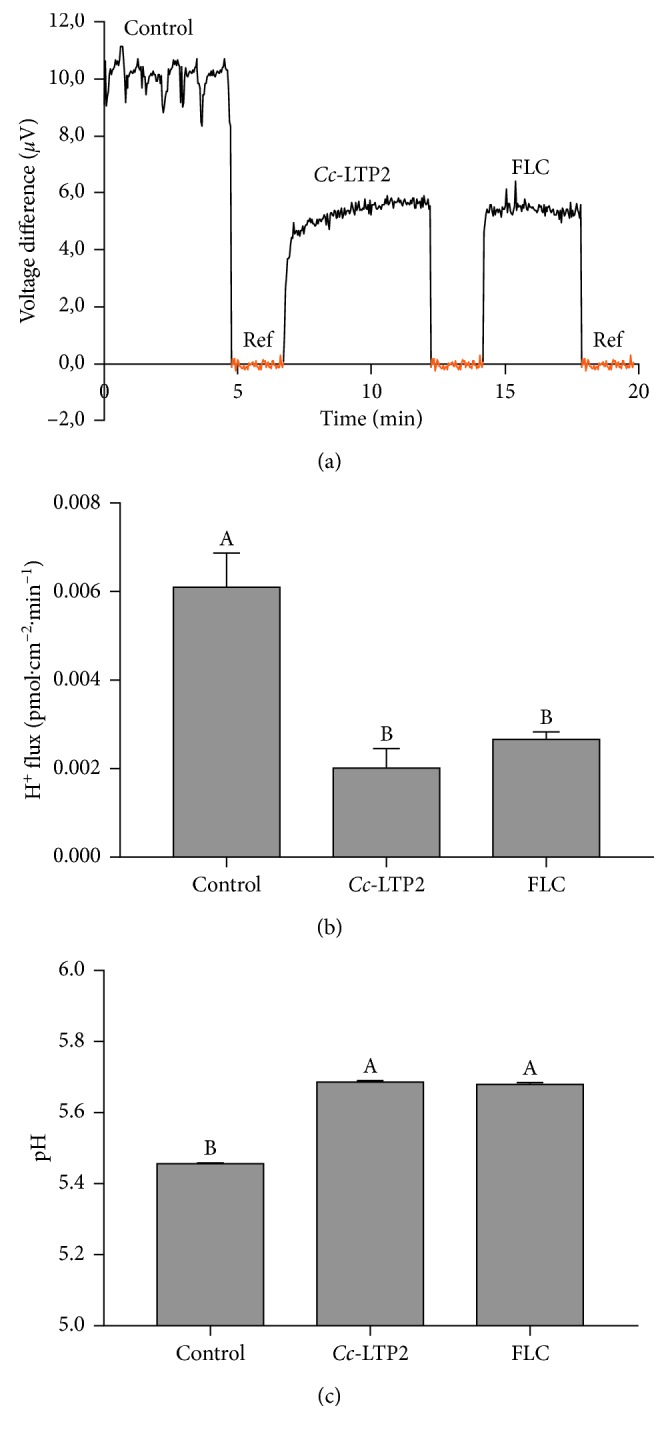
Voltage difference (a), H^+^ efflux rate (b), and root surface pH (c) in *F. solani* cells treated or not (control) with *Cc-*LTP2 (400 *µ*g/mL) or *Cc*-LTP2 (400 *µ*g/mL) + FLC (20 *µ*g/mL). For the H^+^ efflux and pH data, the means are significantly different by Student's *t*-test at *p* ≤ 0.01. Ref represents the background reference.

Extracellular voltage difference, proton fluxes, and surface pH were measured in *F. solani* fungus using an H^+^-selective vibrating probe. A stable H^+^ voltage difference was recorded in the presence or absence of *Cc*-LTP2 peptides and *Cc*-LTP2 + FLC (Figure 5(a)). Before the exposure to 400 *µ*g/mL of *Cc*-LTP2, the fungal cells showed a steady-state extracellular H^+^ efflux activity of 6.09 ± 0.71 pmol·cm^−2^·min^−1^, while after treatment with the peptide, the H^+^ efflux showed an inhibition of approximately 67% (*p* ≤ 0.0001, Tukey test) (Figure 5(b)). After the addition of FLC (20 *µ*g/mL), fungal cells had H^+^ effluxes of approximately 2.66 ± 0.93, showing that inhibition was not enhanced by the combination *Cc*-LTP2 + FLC (*p*=0.1198, Tukey test) (Figure 5(b)). Consequently, the colony surface pH increased significantly when the fungal cells were exposed to *Cc*-LTP2 peptides (Figure 5(c)). However, 10 min after the removal of *Cc*-LTP2 peptides from the medium, the basal H^+^ efflux and surface pH returned to normal, suggesting that the inhibition is *Cc*-LTP2 peptide-dependent (data not shown).

## 4. Discussion

Diseases and pests that attack crops are responsible for large annual losses from the planting to storage, indicating a major threat to global food security. Pathogenic microorganisms such as viruses, fungi, and bacteria are responsible for a decrease of more than 10% in global food production [37]. Antimicrobial peptides (AMPs) are emerging as a possible alternative to help combat microorganisms that have become increasingly resistant to drugs used commercially. These molecules have been isolated from different species from bacteria to vertebrates and are usually part of the innate immune systems of these organisms, where they play an important role in host defense against infection [38, 39].

LTPs are among the many classes of peptides with antimicrobial activity that have been isolated from plants. They exhibit antimicrobial activity against various microorganisms, including viruses [40], bacteria [21], and fungi [20, 22]. GRP has been described to be active against bacteria [27], filamentous fungi [24, 25], and yeasts [26]. The present work was designed to study the antimicrobial activity and the mechanism of action of these two peptides that represent the following classes: LTP (*Cc*-LTP2) and GRP (*Cc*-GRP). One of the major problems in combating fungal infections is the large number of pathogens that are becoming increasingly resistant to classical antibiotics such as fluconazole (FLC), which is used commercially to combat fungal infection [41]. A potential alternative that may be used is the combination of antimicrobial molecules such as AMPs with classic drugs such as FLC.

Therefore, in this study, to enhance the effects of *Cc*-LTP2 and *Cc*-GRP peptides through possible synergistic activity, they were combined with nontoxic concentration of FLC. Analyzing the dry weight of the fungus, we observed a significant reduction in fungal growth and changes in the growth pattern (Figure 1). Therefore, in the absence and presence of the combinations *Cc*-LTP2 + FLC and *Cc*-GRP + FLC, we observed a significant reduction in dry weight of the fungus that was grown in the presence of the peptides in combination with a nontoxic concentration of the FLC. This showed that these peptides can act synergistically with FLC.

Results reported by Taveira et al. [31] showed synergistic effects between FLC and the *Ca*Thi (AMP from *Capsicum annuum* fruits), a peptide belonging to thionin family, against six yeasts of medical importance, and this combination caused drastic morphological changes in the cells of these yeasts. Similar synergistic results for *Ca*Thi were also observed for the fungus *F. solani* when combined with FLC [42]. The combinations *Cc*-LTP2 + FLC and *Cc*-GRP + FLC also promoted various morphological changes in *F. solani* cells, such as a reduction in the number of hyphae and disorganized and retracted cytoplasm (Figure 2). In the literature, different compounds have been described to act synergistically with FLC. Silva et al. [43] demonstrated the synergistic action of a flavonoid in combination with FLC on the growth of the yeast *Candida tropicalis*. Zhai et al. [44] showed that the combination of a classical antibiotic, polymyxin B (polypeptide antibiotics obtained from different species of *Paenibacillus polymyxa*), with FLC can be an alternative to combat systemic cryptococcosis. The Rs-AFP1 and Rs-AFP2 (AMPs from *Raphanus sativus* seeds belong to the plant defensin family) were observed to act synergistically with caspofungin to inhibit the *Candida albicans* biofilm [45].

Many AMPs have amphipathic characteristics, which confer them with the ability to interact with biological membranes. This interaction may lead to disorganization of the membrane and increased permeabilization, including the formation of pores, which may or may not be related to the antimicrobial activity of the peptide and the death of the microorganism [46]. To assess whether the *Cc*-LTP2 and *Cc*-GRP peptides separately and in combination with FLC could permeabilize the membrane of the fungus *F. solani*, the Sytox Green dye, which penetrates only cells that have compromised membranes, was used. *Cc*-GRP applied separately or in combination with FLC was able to permeabilize the membrane of *F. Solani.* However, *F. solani* cells grown in the presence of *Cc*-LTP2 (400 *µ*g/mL) and FLC (20 *µ*g/mL) separately, as well as in the control medium, showed no fluorescence except when the two compounds were combined. Thus, the two compounds work synergistically to permeabilize the membrane of the fungus (Figure 3).

The results observed in this study show that peptides can modulate the influx of ions through the membrane by modifying the membrane permeability. This phenomenon has been observed for several proteins and peptides isolated from different plants, including those from coffee. Zottich et al. [23] isolated a peptide named “*Cc*-LTP” from *Coffea canephora* seeds, and it was able to permeabilize the membranes of different yeasts such as *C. albicans*, *C. tropicalis*, and *Saccharomyces cerevisiae.* The *Cc*-GRP evaluated in this study was previously isolated by Zottich et al. [26] and was shown to inhibit the growth of *C. albicans* and *C. tropicalis* and to increase their membrane permeability. A combination of other compounds with FLC may also cause changes in the membranes of microorganisms. An example is glabridin, a secondary metabolite that acts synergistically with FLC to cause damage to the cell envelope of *C. albicans* and *C. tropicalis*, thereby potentiating the antifungal effects of FLC [41].

The action of AMPs on the membrane of microorganisms can change the flow of protons, causing an imbalance in homeostasis and may lead to the microorganism's death [32]. The H^+^ flux across the plasma membrane plays an essential role in the physiology of the fungal cell. The H^+^ flux is usually mediated by the H^+^-ATPase pump, and interference with this flux can lead to cell death. These effects were monitored by measuring the flow of protons in the *F. solani* cells. The results presented herein show that, after treatment with *Cc*-LTP2 separately and in combination with FLC, an efflux of H^+^ resulted in a significant inhibition of growth by approximately 67%, demonstrating that this peptide can cause an imbalance in the homeostasis of H^+^ (Figure 5). Similar results were reported by Diz et al. [47] and Ribeiro et al. [48], in which peptides present in fractions that were obtained from *Capsicum annuum* seeds completely inhibited the efflux of H^+^ in *S. cerevisiae* (100% inhibition).

Some studies have shown that an increase in ROS production is a mechanism employed by many AMPs [49]. ROS are reactive molecules generated as metabolic products of endogenous or exogenous sources. These molecules from oxygen intracellular metabolism can act on the activation of transcription factors through signal transduction [50]. Within cells, ROS molecules are normally in equilibrium with antioxidants; however, when this critical balance is interrupted, there is excessive production of ROS, resulting in significant cellular damage due to oxidative stress [51]. When examining whether *Cc*-LTP2 and *Cc*-GRP separately or in combination with FLC were able to induce the endogenous production of ROS, it was found that the *Cc*-LTP2 and FLC alone did not induce the production of ROS; this was observed only when these two compounds were combined. *Cc*-GRP both separately and in combination with FLC was able to increase the endogenous production of ROS (Figure 4). Silva et al. [43] showed that a flavonoid when combined with FLC induced the exposition of phosphatidylserine, an important marker of apoptosis, and induced endogenous production of ROS. Similar results were also observed when the compounds were used separately.

Many studies have attempted to combine classical antifungals such as FLC and amphotericin B with different compounds to combat resistant organisms. However, such studies have been mainly developed in the area of human pathogens. There were no studies similar to the one conducted in this work to attempt to combat pathogenic fungi that have become increasingly resistant, mainly representatives of the genus *Fusarium* which are the main pathogens of agronomic importance.

## 5. Conclusion

The genus *Fusarium* is a group of filamentous fungi most studied and contains some of the most important species of plant pathogens economically affecting agriculture and horticulture. Another concern is that a large number of *Fusarium* species are able to cause infections not only in plants but also in animals and humans. Therefore, the discovery of new antifungal agents is of paramount importance. Here, we demonstrated that *Cc*-LTP2 and *Cc*-GRP combined with fluconazole (FLC) have antifungal effects against *F. solani*, and they work by permeabilizing the membrane and inducing oxidative stress response in this fungus. Our results show that the combined treatment of *Cc*-LTP2 and *Cc*-GRP with FLC is a strong candidate for studies aimed at improving ways of combating *F. solani*, and this strategy is even more interesting because it can minimize selection of resistant microorganisms.
